# Comparison of the Sedative, Recovery, and Analgesic Effects of Oral Dexmedetomidine, Ketamine, and Midazolam for Premedication in Children Undergoing Inguinal Hernia Surgery: A Randomized Double-Blind Study

**DOI:** 10.5812/aapm-166505

**Published:** 2025-12-31

**Authors:** Mohamed Zakarea Wfa, Motaz Amr Abu Sabaa, Hadal Hassan Mohsen, Laila Elahwal

**Affiliations:** 1 Faculty of Medicine, Tanta University, Tanta, Egypt; 2 Department of Anesthesiology, and Surgical Intensive Care, Faculty of Medicine, Ain Shams University, Cairo, Egypt

**Keywords:** Oral Dexmedetomidine, Ketamine, Midazolam, Premedication, Pediatric Anesthesia

## Abstract

**Background:**

Midazolam is widely used for its anxiolytic and amnestic effects, while dexmedetomidine provides sedation and analgesia, and ketamine induces sedation, analgesia, and amnesia. Oral administration is commonly accepted in children undergoing inguinal hernia surgeries.

**Objectives:**

This study aimed to examine oral dexmedetomidine, ketamine, and midazolam as premedication in children having inguinal hernia operations.

**Methods:**

This randomized, double-blind study included 60 children (ages 3 - 7, ASA I-II) who underwent inguinal hernia operations. Subjects were randomly assigned to 3 groups: Group K received oral ketamine at 6 mg/kg, group D received oral dexmedetomidine at 4 µg/kg, and group M received oral midazolam at 0.5 mg/kg.

**Results:**

No substantial differences were seen in the five-point sedation score (P = 0.2811) or the Parental Separation Anxiety Scale (PSAS; P = 0.1122). Group D had a markedly reduced recovery time in comparison to groups M and K (P < 0.001). Furthermore, group D exhibited markedly reduced face, legs, activity, cry, and consolability (FLACC) scores at 15 and 20 minutes compared with groups M and K (P = 0.001, 0.016, respectively).

**Conclusions:**

Oral dexmedetomidine, ketamine, and midazolam provided comparable sedation quality and parental separation ease in children undergoing inguinal hernia surgeries. However, dexmedetomidine showed significant advantages by reducing recovery time and improving postoperative pain scores, despite a higher incidence of bradycardia in some patients. Thus, oral dexmedetomidine appears to be an efficient and safe alternative for pediatric premedication, provided careful heart rate monitoring.

## 1. Background

Over 60% of children have difficulty achieving relaxation preceding surgery (1). Infants and preschoolers may exhibit understandable discomfort when detached from their parents for medical procedures necessary before surgery, such as vein puncturing or mask placement (2). Uncontrolled anxiety can lead to difficult induction, elevated pain following surgery, increased analgesic demands, restlessness during emergence, and possible postoperative psychological and behavioral complications (3).

The preoperative phase is the most stressful for preschool children who undergo surgery. Their detachment from their parents and their apprehension towards medical practitioners and the injection of needles heighten their preoperative worry (4). Several diagnostic procedures, multiple needle sticks, blood draws, and pharmacological treatments such as chemotherapy can cause psychological discomfort in children with cancer (5). Increased heart rate, blood pressure, and cardiac excitability are the results of the parasympathetic, sympathetic, and endocrine systems being stimulated by the acute stressor that is anxiety before surgery (6).

Furthermore, they are likely to exhibit delirium, sleep problems, and behavioral alterations (7). Midazolam, clonidine, dexmedetomidine, and ketamine are among the various pharmacological medications that have been proposed for use as sedative premedication to alleviate anxiety prior to surgery and to provide a smooth departure from parents (8). Midazolam, a GABA receptor agonist, is the most commonly employed medication for pediatric preoperative management due to its amnestic and anxiolytic properties (9, 10).

Dexmedetomidine has calming and analgesic effects on the central nervous system; it is a highly selective alpha-2 adrenergic agonist (11). Sedation, anesthesia, weakness, analgesia, and forgetfulness are some of the effects of ketamine, an N-methyl-d-aspartate (NMDA) receptor antagonist (12). There was a lot of statistical variation in the degree of sleepiness induced by oral midazolam, as well as in the means of systolic blood pressure, heart rates, parental separation, and mask acceptance (13, 14).

There is a wide range of patient acceptance when it comes to the various routes of administration for sedative premedication in pediatric patients, including oral, rectal, sublingual, and intranasal. One simple method of drug administration is inhalation of nebulized medicine; it is quick to set up, does not necessitate venipuncture, and is linked to high drug bioavailability (15).

## 2. Objectives

The objective of this work has been to examine oral dexmedetomidine, midazolam, and ketamine as premedication in pediatric cases having inguinal hernia operations.

## 3. Methods

This randomized, double-blind comparative work involved sixty children who underwent inguinal hernia surgeries and were admitted to Tanta University hospitals over the period from March 2025 to September 2025, after approval from the ethical committee (approval code: 36264PR1102/2/25), with clinical trial approval number (NCT06879496).

Written informed approval was obtained from these children's parents. The study aim was explained to them, and they were assigned confidential code numbers.

Any unexpected risks that arose during the study were immediately communicated to the children’s parents and the ethical committee.

The criteria for inclusion in this study were children aged 3 to 7 years, both sexes, with an American Society of Anesthesiologists (ASA) physical status of I-II. Non-inclusion criteria included parental refusal to participate and any documented allergy to the study medications. Exclusion criteria applied to participants who were initially eligible but later met any of the following conditions: Substantial organ dysfunction, cardiac dysrhythmia, current use of psychiatric medications, or intellectual disability.

### 3.1. Randomization and Blinding

Every patient's code was securely saved in an opaque envelope, and a random list was constructed using an internet randomization application (http://www.randomizer.org). The patients were divided into three equal groups using an allocation ratio of 1:1:1: Group K received oral ketamine at 6 mg/kg, group D received oral dexmedetomidine at 4 µg/kg, and group M received oral midazolam at 0.5 mg/kg.

The study drugs were prepared by a member of the research team (one of the authors) who was not involved in patient care, drug administration, anesthesia, or outcome assessment. The calculated doses of dexmedetomidine, ketamine, or midazolam were mixed with a fixed volume of apple juice and labeled only with the patient code to ensure identical appearance, taste, color, and volume. Children, their parents, anesthesiologists involved in anesthesia induction and intraoperative care, personnel administering the oral premedication, and outcome assessors were all blinded to group allocation.

### 3.2. Study Protocol

Prior to the operation, group M was administered an oral dosage of 0.5 mg/kg midazolam (not exceeding 15 mg), group D received 4 µg/kg of dexmedetomidine, and group K was given 6 mg/kg of ketamine.

All premedication drugs were prepared immediately before administration. The calculated doses of dexmedetomidine (4 µg/kg) or ketamine (6 mg/kg) were drawn from commercially available stock solutions using a sterile syringe. Each dose was then mixed with exactly 5 mL of apple juice to standardize the final administered volume across all participants in the preoperative holding room 40 minutes before the onset of anesthesia.

Because the administered volume was fixed at 5 mL for all patients, the final concentration of the mixture varied according to each child's weight-based dose, while the total oral volume remained constant. The drug-juice mixture was gently inverted to ensure homogeneity, and it was administered within 5 minutes of preparation to maintain stability and prevent degradation.

Afterwards, a systemic anesthetic was given. Typical monitoring procedures were electrocardiography (ECG), measuring end-tidal carbon dioxide, continuously monitoring arterial oxygen saturation, pulse oximetry, and non-invasively assessing blood pressure every 5 minutes. In every instance, the anesthetic technique remained unchanged. Anesthesia was initiated with a Jackson-Rees breathing circuit that contained 8% sevoflurane in 100% oxygen. Following the injection of an anesthetic, an intravenous cannula was placed

The next step was to install a laryngeal mask airway after administering 1 mg/kg of intravenous propofol to the participants. A combination of 50% oxygen and 50% air was used to sustain anesthesia with sevoflurane. No more sedatives or opioids were used, and spontaneous breathing was maintained during the whole procedure. Following the child's voluntary airway maintenance being confirmed and the absence of hemodynamic instability, the laryngeal mask was removed and the child was taken to the post-anesthesia care unit following the operation.

Measurements of the Face, Legs, Activity, Cry, and Consolability (FLACC) Scale for pain and Emergence Agitation (EA) Scale were recorded for one hour. Following an Aldrete-Kroulik recovery score above 9, those individuals were transferred to the ward.

Perioperative complications were observed and documented, including hypotension, bradycardia, and emesis. Fluid bolus administration was provided for hypotension [(a 20% decrease in basal mean arterial pressure (MAP)]. Bradycardia is characterized by a heart rate of less than 60 beats per minute, necessitating the use of atropine.

### 3.3. Assessment Parameters

Preoperative assessments: The heart rate, non-invasive blood pressure, and respiratory rate were evaluated at baseline (0 min) and at 5, 10, 20, and 30 minutes after the conclusion of the trial and medication delivery.

The level of sedation was measured at the previously mentioned intervals using a five-point Sedation Scale: 1 = agitated, 2 = conscious, 3 = relaxed, 4 = dizzy, 5 = asleep (16). A score of 3 or above was considered appropriate for sedation.

The drug's acceptability among participants was assessed using a four-point Scale as described below: 1 = excellent, received medication without complaints; 2 = acceptable, expressed dissatisfaction, briefly distressed or upset, but subsequently took medication; 3 = moderate, expressed complaints, initially resistant but finally agreed to treatment; 4 = unsatisfactory, refused medication (17).

At the end of the preoperative period, the effects of separation from parents were assessed using a four-point Parental Separation Anxiety Scale (PSAS) as shown below: 1 = simple separation; 2 = produces whimpers but is easily comforted and not dependent; 3 = cries and is challenging to assist, however is not attached to parents; 4 = cries and attaches to parents. Parental Separation Anxiety Scale scores of 1 and 2 indicated adequate separation, whereas ratings of 3 and 4 indicated difficult separation (18).

Intraoperative assessments: The child's sedative level was evaluated upon arriving at the operating room (OR). The acceptability of the anesthetic mask by patients was assessed using a four-point Mask Acceptance Scale (MAS) as follows: 1 = excellent, fearless, cooperative, readily accepts mask; 2 = good, little apprehension towards mask, easily reassured; 3 = fair, considerable apprehension towards mask, not soothed by reassurance; 4 = poor, scared, sobbing, or belligerent (19).

Heart rate and blood pressure were measured at baseline (0 minutes) and at 5, 10, 15, and 20 minutes after the onset of general anesthesia. The duration of anesthesia and recovery time (the interval from the cessation of sevoflurane until the sedation score returned to baseline) was documented in minutes.

Hypotension was managed with an intravenous fluid bolus of 10 mL/kg crystalloid, while bradycardia (heart rate < 60 beats/min) was treated with intravenous atropine at a dose of 0.02 mg/kg when clinically indicated.

Early postoperative assessments: Heart rate and blood pressure were monitored at admission to the post-anesthesia care unit (0 minutes, baseline) and then at 15, 30, 45, and 60 minutes. Recovery was evaluated using the Three-Point EA Scale, as explained: 1 = calm; 2 = agitated but compliant with verbal directives; and 3 = confrontational and confused. A score of 2 or above indicated sevoflurane-associated emerging agitation (16).

The FLACC Scale was used to assess pain severity, with a maximum score of 10 (20). All patients received paracetamol 15 mg/kg as routine analgesia.

### 3.4. Measurements

The recorded and collected data included demographic characteristics (age, weight, ASA class, and duration of surgery), hypotension, decreased heart rate, and vomiting, hemodynamic parameters (MAP and heart rate), five-point sedation score, PSAS, postoperative FLACC Scale, and any adverse effects.

The primary endpoint of this current work was the five-point sedation score evaluated upon arrival in the OR, forty minutes post-drug delivery. The secondary objectives included assessing the separation from Parents Anxiety Scale, hemodynamic parameters, recovery duration, postoperative pain measured by the FLACC Scale, and observing adverse effects, including bradycardia and hypotension.

### 3.5. Sample Size Calculation

The sample size was determined using G*Power 3.1.9.2 (University of Kiel, Germany). A pilot study was performed with five subjects per group, revealing that the mean ± standard deviation (SD) five-point sedation scores were 4.20 ± 1.79 for dexmedetomidine, 3.20 ± 1.64 for ketamine, and 2.20 ± 1.58 for midazolam. The sample size was determined by the following factors: An effect size of 0.517, a 95% confidence interval, 90% statistical power, a 1:1:1 group ratio, and the inclusion of three additional cases per group to account for dropout. Consequently, we recruited 20 subjects per group.

### 3.6. Statistical Analysis

Statistical analysis was performed using SPSS version 27 (IBM^©^, Chicago, IL, USA). The Shapiro-Wilk test and histograms were utilized to evaluate the normality of the data distributions. Quantitative parametric results were presented as mean and SD and evaluated using the ANOVA (F) test followed by a post hoc Tukey test. Quantitative nonparametric data were presented as median and interquartile range (IQR) and analyzed using the Kruskal-Wallis test, while the Mann-Whitney test was used for comparisons between groups. Qualitative parameters were represented as frequencies and percentages (%) and analyzed using the chi-square test. A two-tailed P-value below 0.05 was considered to be substantial.

## 4. Results

**Figure 1.**
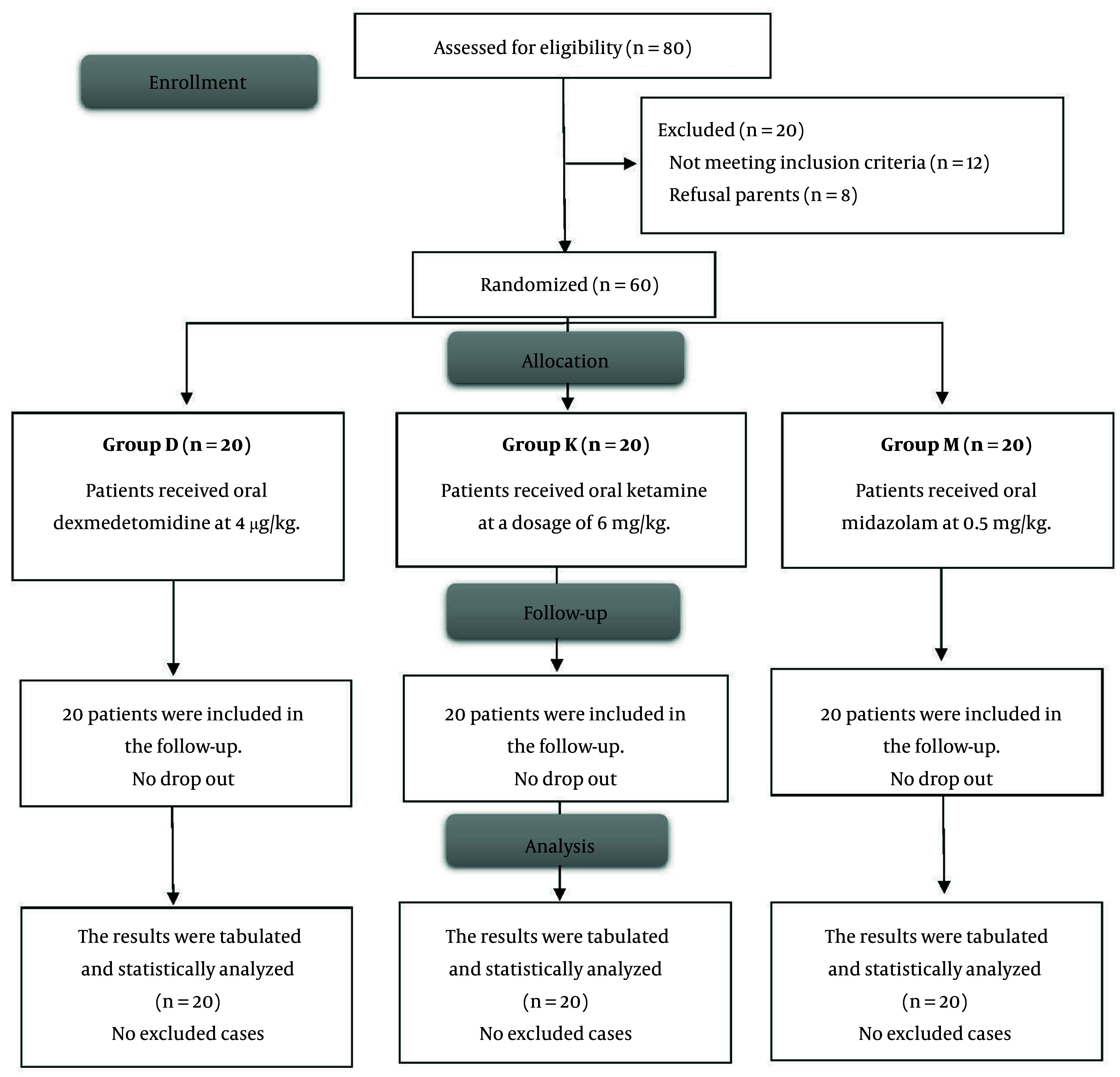
Flowchart of patient enrollment, randomization, and follow-up

Twelve individuals were deemed ineligible for the trial, while the guardians of eight patients opted not to participate. Sixty (60) patients were then allocated into three groups, including 20 individuals each. All patients were monitored and assessed statistically (Figure 1). 

The demographic data showed no significant differences among the three groups. Group D had a notable disparity in heart rate, as three individuals experienced bradycardia. Nonetheless, there was no notable disparity in blood pressure among the three groups (Table 1). 

**Table 1.** Demographic Data and Clinical Characteristics of Patients in the Dexmedetomidine, Ketamine, and Midazolam Groups ^a^

Variables	Groups ^b^			Chi-square	P-Value
	D	K	M		
**Age (y) **	4.8 ± 1.36	4.9 ± 1.25	4.75 ± 1.45	-	
**Sex ratio (M/F)**	11/9	8/12	12/8	0.4199	
Male	11 (55)	8 (40)	12 (60)		
Female	9 (45)				
**ASA status**				0.7881	
ASA I	14 (7)	15 (75)	13 (65)		
ASA II	6 (30)	5 (25)	7 (35)		
**Weight **	18.55 ± 4.097	17.8 ± 3.806	17.6 ± 4.285	-	
**Duration of surgery (min)**	42.75 ± 11.41	43 ± 10.52	42.75 ± 11.41	-	
**P-value**				-	
Age	-	0.810	0.911		0.728 ^c^
Weight	-	0.552	0.478		0.877 ^c^
Duration of surgery (min)	-	0.9454	0.999		0.9479 ^c^

Abbreviations: P-value, probability value; ASA, American Society of Anesthesiologists.

^a^ Values are expressed as mean ± SD or No. (%).

^b^ Group D: Dexmedetomidine group, Group K: Ketamine group, Group M: Midazolam group.

^c^ P-values indicate comparisons between groups K and M.

No substantial variation was seen in the five-point sedation score (P-value = 0.2811). No substantial change was seen on the PSAS (P-value = 0.1122). Time from drug prescription to separation from parents was significantly lower in group D than in groups M and K, and in group K than in group M (P-value < 0.001). Recovery time in group D was significantly shorter than in groups M and K (P-value < 0.001). The FLACC score was significantly lower in group D compared with groups M and K at 15 minutes and 20 minutes (P < 0.001, 0.0160, respectively), while there was no significant difference at 5 min, 10 min, 25 min, or 30 min among the three groups, suggesting a transient postoperative analgesic effect (Table 2). 

**Table 2.** Postoperative Outcomes, Sedation Scores, Parental Separation Anxiety, Recovery Time, and Face, Legs, Activity, Cry, and Consolability Pain Scale Among Patients in the Dexmedetomidine, Ketamine, and Midazolam Groups ^a^

Outcome/Scale	Groups ^b ^			P-Value/Chi-square
	D	K	M	
**Postoperative complications**				
Vomiting	0 (0)	3 (15)	5 (25)	0.0645
Hypotension	2 (10)	0 (0)	0 (0)	0.1263
Bradycardia	3 (15)	0 (0)	0 (0)	0.0425
**Sedation score (Five-Point Scale)**				
Good sedation	11 (55)	10 (50)	12 (60)	0.2811
Poor sedation	9 (45)	8 (40)	8 (40)	
Unresponsiveness	0 (0)	2 (10)	0 (0)	
**Separation anxiety**				
Acceptable	17 (85)	15 (75)	18 (90)	0.432
Nonacceptable	3 (15)	5 (25)	2 (10)	
**Time from drugs prescription to separation of parents (min)**	5.2 ± 0.83	30.8 ± 3.4	37.3 ± 4.94	< 0.001 ^c^ (group D vs. others) and (group K vs. group M)
**Recovery time (min)**	20.4 ± 3.7	34.7 ± 6.6	38.4 ± 5.1	< 0.001 ^c^ (group D vs. others), 0.059 (group K vs. group M)
**FLACC Pain Scale (median range), (min)**				
5	0 - 1	0 - 1	0 - 1	1.000
10	0 - 1	0 - 2	0 - 1	0.0699
15	0 - 1	1 - 3	0 - 1	< 0.001 ^c^
20	0 - 2	1 - 3	1 - 2	0.0160
25	1 - 2	1 - 3	1 - 2	0.0622
30	1 - 2	1 - 3	1 - 2	0.0622

Abbreviations: P-value, Probability value; FLACC, face, legs, activity, cry, and consolability.

^a^ Values are expressed as mean ± SD or No. (%).

^b^ Group D: Dexmedetomidine group, Group K: Ketamine group, Group M: Midazolam group.

^c^ Statistically significant (P-value < 0.05).

## 5. Discussion

Our study aimed to compare the effectiveness of oral dexmedetomidine, ketamine, and midazolam as premedication in children undergoing inguinal hernia surgery.

In this randomized double-blind study, oral dexmedetomidine, ketamine, and midazolam provided comparable levels of preoperative sedation and facilitated parental separation in children undergoing inguinal hernia surgery. However, dexmedetomidine was associated with a significantly shorter recovery time and lower postoperative pain scores compared with ketamine and midazolam. Although bradycardia occurred more frequently in the dexmedetomidine group, it was transient and did not require pharmacological intervention.

Despite the wide use of midazolam, dexmedetomidine, and ketamine as pediatric premedicants, there is a clear lack of studies directly comparing all three agents simultaneously using the oral route in a standardized surgical setting (21).

The three groups had comparable demographic profiles and similar amounts of operating time. Bradycardia and hypotension occurred in 5 patients in group D (3 cases suffered from bradycardia and 2 suffered hypotension), with a tolerable range that did not need intervention.

Consistent with our findings, Singh et al. (22), compared premedication using oral dexmedetomidine and oral ketamine, revealing that oral dexmedetomidine at a dosage of 3 - 5 µg/kg resulted in a dose-dependent decline in heart rate and systolic blood pressure, with a maximum reduction of up to 20% relative to baseline values.

Kumari et al. (23) noted a reduced mean blood pressure in cases administered oral dexmedetomidine compared to those given oral midazolam, assessed 45 - 60 minutes post-administration (65.72 vs. 71.28 mm Hg, P < 0.001). Prabhu and Mehandale (24) documented comparable findings. Significantly, no individuals in the dexmedetomidine cohort needed medical intervention. Nevertheless, Lalin et al. (25) indicated no statistically substantial variations in mean blood pressure or heart rate among cases administered oral dexmedetomidine and those receiving oral midazolam.

The sedation score at thirty minutes was comparable across the three groups in the study we conducted. Approximately 55% of cases in the dexmedetomidine group, 60% in the midazolam group, and 50% in the ketamine group exhibited optimal sedation at the 30-minute point. This aligns with the work of Sajid et al. (26). In contrast to our findings, Kumari et al. (23) documented a more rapid onset and elevated mean sedation ratings at 30, 45, and 60 minutes with oral midazolam. Jannu et al. (27) similarly observed a more rapid onset of drowsiness and a quicker attainment of peak sedative effects in the midazolam group relative to the oral dexmedetomidine group. While we did not study the initiation of sedation, the medications demonstrated comparable efficacy in achieving adequate drowsiness after 30 minutes.

No substantial difference in parental separation anxiety scores at 40 minutes was observed across the three groups, consistent with the findings of Jannu et al. (27). Acceptable scores for parental separation anxiety were seen in 85%, 75%, and 90% of children in the dexmedetomidine, ketamine, and midazolam groups, respectively (P = 0.432). Kumari et al. (23) discovered that oral midazolam is more efficient than dexmedetomidine in facilitating separation from parents, contrary to our outcomes. Mountain et al. (28), however, found no substantial variance in acceptable conduct during parental separation or mask acceptance in children administered dexmedetomidine and midazolam 30 minutes before induction.

According to our study, there is a substantial reduction in recovery time in group D compared with groups M and K (P < 0.001). In addition to our findings, Abdel-Ghaffar et al. (6) demonstrated a substantial reduction in recovery time for the group administered nebulized dexmedetomidine compared to the other two groups receiving nebulized ketamine and midazolam.

According to our findings, Schmidt et al. (29) found no substantial differences among the clonidine, dexmedetomidine, and midazolam groups; nevertheless, the dexmedetomidine group exhibited a recovery time of 28.8 ± 20.4, which was shorter than that of the other two groups.

Surendar et al. (30) reported the same result as ours: A substantial reduction in pain postoperatively in the dexmedetomidine group and the ketamine group versus the midazolam group. The differences between their study and ours are that the route they used was intranasal and that they measured the pain perioperatively.

This study has some limitations, including the use of oral premedication, which may be influenced by variable bioavailability in children. The sample was limited to ASA I-II preschool children undergoing inguinal hernia repair, the single-center design may limit generalizability, and no long-term postoperative or behavioral outcomes were evaluated. Additionally, there was a lack of pharmacokinetic standardization, as a fixed oral volume (5 mL) was used for all children, resulting in weight-dependent variability in drug concentration per milliliter, which may have influenced drug absorption. Future multicenter research with a larger sample size and longer follow-up should evaluate non-oral premedication routes to minimize pharmacokinetic variability, include children across wider ASA classes and multiple surgical procedures, and standardize oral drug concentrations or adjust the administered volume according to body weight to minimize pharmacokinetic variability and better evaluate the clinical effects of oral premedication in children.

### 5.1. Conclusions

Oral dexmedetomidine, ketamine, and midazolam provided comparable sedation quality and parental separation ease in children undergoing inguinal hernia surgeries. However, dexmedetomidine showed significant advantages by reducing recovery time and improving postoperative pain scores, despite a higher incidence of bradycardia in some patients. Thus, oral dexmedetomidine appears to be an efficient and safe alternative for pediatric premedication, with careful monitoring of heart rate.

## Data Availability

The dataset presented in the study is available on request from the corresponding author during submission or after publication.

## Clinical Trial Registration Code:

NCT06879496.
